# Local anesthetics induce autophagy in young permanent tooth pulp cells

**DOI:** 10.1038/cddiscovery.2015.24

**Published:** 2015-09-07

**Authors:** H Zhuang, D Hu, D Singer, J V Walker, R B Nisr, K Tieu, K Ali, C Tredwin, S Luo, S Ardu, B Hu

**Affiliations:** 1 Department of Cariology, Endodontology and Operative Dentistry, Peking University School and Hospital of Stomatology, 22 South Zhongguancun Avenue, Haidian District, Beijing, P.R. China; 2 Peninsula Dental School, Plymouth University Peninsula Schools of Medicine and Dentistry, 16 Research Way, Plymouth, UK; 3 Department of Anesthetics, School of Stomatology, Capital Medical University, 4 Tian Tan Xi Li, Dong Cheng District, Beijing, P.R. China; 4 Peninsula Medical School, Plymouth University Peninsula Schools of Medicine and Dentistry, Research Way, Plymouth, UK; 5 Division of Cariology & Endodontology, Dental School, University of Geneva, Geneva, Switzerland

## Abstract

Pulp cells are essential for tooth development, and dentin repair and regeneration. In addition these cells have been identified as an important stem cell source. Local anesthetics are widely used in dental clinics, as well as the other clinical disciplines and have been suggested to interfere with human permanent tooth development and induce tooth agenesis through unknown mechanisms. Using pig model and human young permanent tooth pulp cells, our research has identified that the local anesthetics commonly used in clinics can affect cell proliferation. Molecular pathway profiling suggested that LC3II is one of the earliest molecules induced by the agents and *p62* is the only common downstream target identified for all the drugs tested. The effect of the drugs could be partially recovered by V-ATPase inhibitor only if early intervention is performed. Our results provide novel evidence that local anesthetics could affect tooth cell growth that potentially can have impacts on tooth development.

## Introduction

Dental treatment involves similar or more frequent use of local anesthesia compared with any other clinical discipline. Local anesthetics are known to work by binding to voltage-gated Na^+^ channels in nerves, therefore block sodium transportation and nerve conduction.^1^ Although the maximum doses of various local anesthetics are established, the side effects of these agents on dental tissues have not yet been fully investigated. The only relevant literature in this regard relates to a canine model, which reported that local anesthetics could accumulate in natural cavities such as the crypts of tooth buds and the mandibular canal.^2^ A recent clinical epidemiological study showed that local anesthesia can potentially interferes with human permanent tooth development and induces tooth agenesis through unknown mechanisms.^3^

Autophagy is a catabolic process involving the degradation of unnecessary or aberrant cellular components through hydrolysis of lysosomes. It therefore controls the turnover of organelles and proteins within cells, and of cells within organisms.^4^ During this process, targeted cytoplasmic constituents are isolated within autophagosomes, which then fuse with lysosomes to form autolysosomes where the cellular material is degraded or recycled.^4^ It was previously observed that anesthesia drugs could induce vacuolation.^5^ However, neither the mechanisms responsible for vacuolation nor its consequence has been reported. Vacuoles have a major role in autophagy and maintain a balance between biogenesis (production) and degradation (or turnover) for many substances and cellular structures.

In this study, using a pig model and *in vitro* human cell culture, we systematically tested the local concentrations of the agents and the cellular effects and molecular interactions of several local anesthetics routinely used in dental clinics.

## Results

### Local anesthetics remain at high concentration in tooth pulp cells after nerve block injection

The 5-month-old pig we used had a mixed dentitions including deciduous teeth and young permanent teeth, as well as developing third permanent molar tooth that are similar to adolescent children. We decided to test five commercial anesthetic drugs that are commonly used in the clinics of the four dental schools involved in this study: articaine-based agents: Ubistesin, Ubistesin forte and Septanest; mepivacaine-based agent: Scandonest and Lidocaine based agent: Xylocaine. Fluorescein-labeled local anesthetics were injected either around mandibular foramen (for lidocaine) for nerve block (Supplementary Figure 1A) or under the mucosa of the mesial buccal and lingual periapical regions of the first molar (for Ubistasin and Scandonest, not shown) for infiltration, exactly simulating clinical situations. It is noticeable that with nerve block, local anesthetics were able to penetrate into developing third molar in a posterior–anterior direction (Supplementary Figure 1B), with a concentration of 19.88±14.19 mM at the posterior site and 8.72±9.43 mM at the anterior site 2 h after injection and could reach to 16.39±8.36 mM and 22.23±17.45 mM respectively after 16 h inside the two proximities of the tooth (Supplementary Figure 1C and E). In contrast, at 2 h, the infiltration injection could reach to very high concentration at the outermost cell layer of the tooth pulp with 49.54±22.57 mM for Ubistesin and 21.16±15.44 mM for Scandonest (Supplementary Figures 1D and E). However, notably the inner layer cells had much lower concentrations of the drugs at 6.67±7.21 mM for Ubistesin and 9.89±10.28 mM for Scandonest (Supplementary Figures 1D and E). Contrary to nerve block methods, in infiltration injection, the drug concentration rapidly decreased after 16 h with the outermost cell layer only held 16.65±10.70 mM for Ubistesin and 9.89±10.28 mM for Scandonest, whereas in the inner cell layer of the tooth pulps the drugs were entirely eliminated (Supplementary Figures 1C and E).

### Local anesthetics affect tooth pulp cell proliferation in a dose-dependent manner

**Figure 1:**
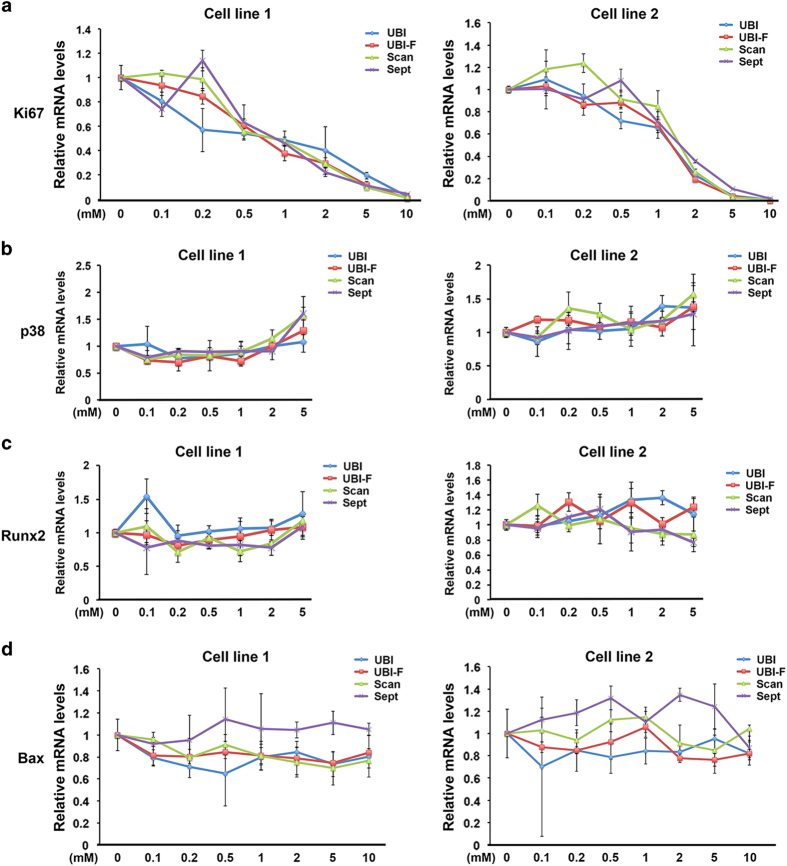
Local anesthetics affect tooth pulp cell proliferation but not differentiation and apoptosis. Real-time RT-PCR analysis of *Ki67* (**a**), *Runx2* (**b**), *p38* (**c**) and *Bax* (**d**) mRNA expression in cells treated with increasing concentrations of the drugs tested for 16 h, normalized to expression of the *36β4* housekeeping gene. TUNEL analysis was performed on the same samples and no differences of apoptosis index have been found (data not shown). UBI, Ubistesin; UBI-F, Ubistesin forte; Scan, Scandonest; Sept, Septanest.

As we found that local anesthetics remained high concentrations particularly in the nerve block method even after 16 h although the test was not performed *in vivo*. We then decided to test the dose effect of the agents on tooth pulp cells by measuring cell proliferation, differentiation and apoptosis, the key parameters that control tooth germ tissue and cell development. At increasing concentrations of the drugs tested, there was a significant dose-dependent reduction of *Ki67* mRNA expression (Figure 1a). In contrast, there was no change in the levels of cell differentiation and cell death upon anesthetic challenge in mRNA expression for the tooth pulp differentiation markers *Runx2* and *p38*, and the apoptosis marker *Bax* at different concentrations (Figure 1b and d, Xylocaine also showed similar regulation pattern of the markers tested, data not shown). The TUNEL assay confirmed that apoptosis was not induced by these drugs (data not shown).

### Local anesthetics induce autophagy in tooth pulp cells

**Figure 2:**
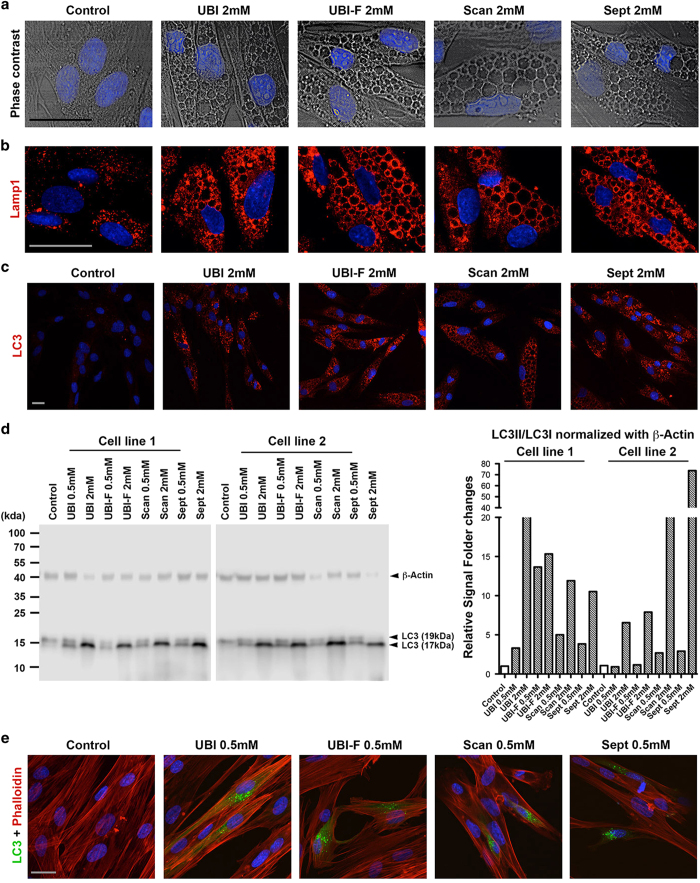
Local anesthetics induce autophagy in tooth pulp cells. (**a**) Phase-contrast microscopy images of drug-treated and control cells. (**b** and **c**) Immunofluorescence analysis of LAMP-1 and LC3 (red) expression in control cells and cells treated with 2 mM anesthetic drug. (**d**) Western blot (left panel) analysis of LC3 expression in two primary dental pulp cell lines with and without drug treatment. Note that LC3 is expressed as two isoforms with molecular weights 17 kDa (LC3-II) and 19 kDa (LC3-I). *β*-Actin was used as a loading control. Signal quantification (right panel) was performed on C-DiGit Blot scanner acquired gel images using Image Studio 4.0 software (LI-COR Biosciences UK Ltd, Cambridge, UK). Relative signal was calculated as LC3II (17 kDa) *versus* LC3I (19 kDa) then normalized against *β*-Actin signal. (**e**) GFP–LC3 fusion protein expression (green) in control cells and cells treated with 0.5 mM anesthetic drug. Cells were counterstained for Phalloidin (red). Cell nuclei were visualized using 4′,6-diamidino-2-phenylindole (DAPI). Additional analysis can be found in Supplementary Figure 2. Scale bars, 20 *μ*m. UBI, Ubistesin; UBI-F, Ubistesin forte; Scan, Scandonest; Sept, Septanest. Scale bars, 10 *μ*m.

In parallel to the decreased cell proliferation, in the presence of all the drugs, increased vacuole formation was also observed in the cytoplasm of dental pulp cells (Figure 2a and data not shown). Immunofluorescence analysis showed that the vacuoles contained LAMP-1 (Figure 2b and Supplementary Figure 2A). As LAMP-1 overexpression is often associated with an accumulation of autophagic vacuoles, we therefore measured the level of autophagy. Autophagosome numbers correlate with the levels of autophagosome-associated protein LC3-II or the number of LC3-positive vesicles.^6^ Immunofluorescence microscopy (Figure 2c and Supplementary Figure 2B) and immunoblotting (Figure 2d) results showed that all anesthetics tested markedly increased levels of LC3, particularly the short half-life form, LC3II (Figure 2d). To provide further evidence of autophagy induction, we analyzed GFP-positive vesicles in cells transfected with GFP–LC3 plasmids. The results showed that GFP production was highly increased in drugs-treated cells, suggesting that all of the local anesthetics tested could induce autophagy (Figure 2e and data not shown). Consistent with our *in vitro* findings, in the local-anesthetic-treated pig mandibles, LC3II was indeed induced in the third molar tooth pulps both at 2 and 16 h after nerve block injection (Supplementary Figure 2C) and in the first molar teeth received infiltration injections, LC3II induction could only be mainly identified at the outermost cell layer of the tooth pulp at 2 h but was quickly removed after 16 h (Supplementary Figure 2D).

### Early autophagy inhibition can reverse anti-cell proliferation effects of local anesthetics on tooth pulp cells

**Figure 3:**
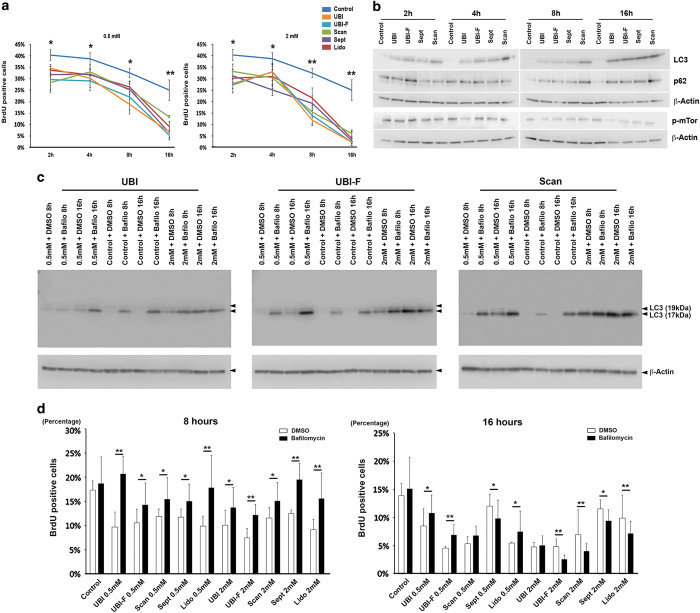
Local anesthetics affect tooth pulp cell proliferation via autophagy induction. (**a**) BrdU labeling analysis in cells treated with 0.5 or 2 mM concentrations of the five anesthetic drugs for 2, 4, 8 and 16 h. (**b**) Western blot analysis of LC3, p62 and mTOR protein levels in cells treated with 2 mM concentration drugs. Similar results have been achieved with lidocaine (data not shown). The quantification of the individual experiment can be found in Supplementary Figures 3A. (**c**) Western blot analysis of LC3 protein levels in cells treated with 0.5 and 2 mM concentrations of the indicated anesthetic drugs and together with 100 nM bafilomycin for 8 and 16 h. *β*-actin was used as a loading control by stripping and reblotting the same blot. Additional experiments and the quantifications of the can be found in Supplementary Figure 3c. (**d**) BrdU-positive cells indexing (four random fields were analyzed under ×10 lens in a Zeiss LSM510Meta laser-scanning microscope, Carl Zeiss Ltd., Cambridge, UK) of the samples from the same experiments indicated in **c** and Supplementary Figure 3B. *P<0.05; **P<0.01.

To understand if the antiproliferative effect of the drugs is time dependent, we performed BrdU labeling analysis of the cells at 0.5 and 2 mM concentrations. The results showed that local anesthetics could already block cell proliferation at 2 h at a rate of twofold change, whereas at 16 h most of the drugs reduced BrdU-positive labeling by more than five times (Figure 3a). Western blotting analysis of LC3, p62 and phosphorylated form mTor suggested that LC3 is the earliest induced molecular by the local anesthetics administration, whereas the induction of p62 and p-mTor only became significant after 8 h (Figure 3b and supplementary Figure 3A), suggesting autophagosome formation is among the earliest cellular reactions upon local anesthetic drug challenge.

Autophagy has been reported to be able to reduce cell proliferation.^7^ We therefore addressed this possibility by treating cells with an autophagy inhibitor, bafilomycin that specifically inhibits vacuolar-type H^+^ ATPase (V-ATPase)^8^ together with each anesthetic drug at 0.5 and 2 mM concentrations for 8 and 16 h. The results showed that at 8 h time point autophagy inhibition did antagonize the antiproliferative effects of anesthetics on tooth pulp cells (Figure 3c and d and Supplementary Figures 3B and C), while at 16 h, bafilomycin still had the rescuing effects in the 0.5 mM agent treated group but failed to do so in the 2 mM group (Figure 3c and d and Supplementary Figures 3B and C).

### p62 is the key target of local anesthetics

**Figure 4:**
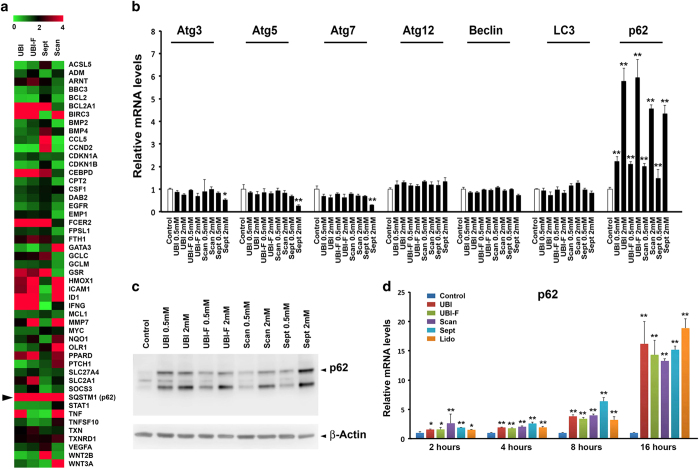
*p62* is the common downstream target of anesthetic drugs on tooth pulp cells. (**a**) Heatmap analysis of PCR array data showing changes in gene expression in tooth pulp cells after 16 h treatment with indicated 2 mM anesthetic drugs (for 0.5 mM dosage, please see Supplementary Figure 4). Colors represent fold changes in expression, as shown in the key. (**b**) Real-time RT-PCR analysis of a panel of autophagy-related genes in primary tooth pulp cells using specific primers, normalized to *36β4* housekeeping gene expression. Error bars represent standard deviation of the triplicate experiments. (**c**) Western blot analysis of p62 expression in drug-treated cells. *β*-actin was used as a loading control by stripping and reblotting the same blot. p62 signal quantification was calculated with normalization against *β*-actin signal (right panel). Similar results have been achieved with lidocaine both for western blot and gene profiling (data not shown). UBI, Ubistesin; UBI-F, Ubistesin forte; Scan, Scandonest; Sept, Septanest. *P<0.05; **P<0.01. (**d**) Real-time RT-PCR analysis of *p62* gene in tooth pulp cells treated with the five anesthetics for different time periods, normalized to *36β4* housekeeping gene expression. Error bars represent standard deviation of triplicate experiments.

To investigate which downstream molecular signaling pathways mediate the effects of local anesthetics on tooth pulp cells, we analyzed the changes in gene expression using the PCR array (PathwayFinder) assay. *p62* was the only gene to consistently show dose-dependent changes in expression for all drugs tested and at different concentrations (Figure 4a and Supplementary Figure 4). Interestingly, transcriptional analysis of a panel of key autophagy genes (including *LC3*) showed *p62* was the only common gene changed by all the drugs tested (Figure 4b). Upregulation of *p62* mRNA and protein was validated by real-time RT-PCR and western blot analysis, respectively (Figure 4b and c). Importantly, *p62* mRNA and protein levels appeared to be linked with local anesthetic concentrations (Figure 4c). In the same time-course study (Figure 3a and b), we also found that *p62* mRNA could be already induced 2 h after the application of the drugs (Figure 4d) but the protein level changes only happened at 8 and 16 h (Figure 3b), suggesting the upregulation of p62 was the subsequent effect of LC3II induction.

### Local anesthetics-induced cell growth arrest is not linked with mitochondrial dysfunctions

**Figure 5:**
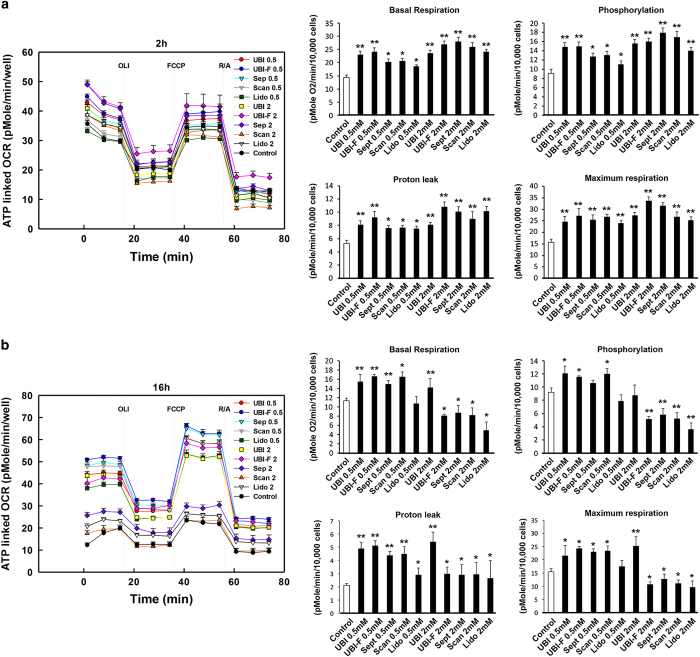
Local anesthetics affect tooth pulp cellular energetics. Key parameters of mitochondrial function including basal respiration, phosphorylation, proton leak and maximum respiration were tested by directly measuring the oxygen consumption rate (OCR) of cells treated with the five anesthetics with 0.5 and 2 mM concentrations for 2 h (**a**) and 16 h (**b**) (8, 16 h results can be found in Supplementary Figure 5). Note that at 16 h basal respiration, phosphorylation and maximum respiration were all reduced in the 2 mM UBI-F, Sept, Scan and Lido treated cells. Lido, Lidocaine; UBI, Ubistesin; UBI-F, Ubistesin forte; Scan, Scandonest; Sept, Septanest. Number abbreviations after individual drug abbreviation: 0.5: 0.5 mM; 2: 2 mM. *P<0.05; **P<0.01.

Local anesthetics have been known to be able to induce cellular stress and autophagy that has been linked with mitochondrial dysfunctions. We therefore measured dynamic cellular energetics using the Seahorse XF reader in cells received local anesthetics challenge. The results showed unexpectedly that at time points 2, 4 and 8 h, all the parameters tested including basal respiration, phosphorylation, proton leak and maximum respiration were induced in the agents treated cells for most of the cases (Figures 5a and b and Supplementary Figures 5A and B) with the exception of the 16-h time point at 2 mM but not 0.5 mM concentration four out of five drugs tested reduced basal respiration, phosphorylation and maximum respiration but not proton leak (Figure 5a and b and Supplementary Figures 5A and B). These results indicate that mitochondrial functions were disturbed only at high concentration (2 mM) after 16 h.

## Discussion

Very little has been known about the impact of local anesthetics on the tooth, particularly on young tooth pulp cells. Articaine, mepivacaine and lidocaine are the most prevalent injectable local anesthetic agents not only in dental clinics but also in other practices. Among them, articaine has been used as the first choice by most of the dentists as it has been proven to be more efficient than the other drugs such as lidocaine due to better nerve block and tooth pulpal anesthesia results.^9^^,^^10^ Articaine contains a thiophene ring and mepivacaine and lidocaine contains a benzene ring that can increase drug lipid solubility. Local anesthetics are known to work by binding to voltage-gated Na^+^ channels in nerves, therefore block sodium transportation and nerve conduction.^1^ To our knowledge, this is the first systematic analysis of the effects for these agents on tooth pulp cell activities. The aim of our study was not to compare the similarities and differences between the agents, but rather we wanted to investigate on common effects of the drugs that potentially could explain the clinically observed increased tooth agenesis ratio in patients who receive local anesthesia treatment^3^ and we have found that the drugs tested could inhibit tooth pulp cells proliferation *in vitro*, in a dosage dependent manner.

It has been reported that local anesthetics such as bupivacaine can uncouple mitochondrial oxygen consumption and ATP synthesis and reduce ATP synthesis.^11^ Mepivacaine can also inhibit mitochondrial respiration^12^ and lidocaine can cause mitochondrial injury and induce apoptosis in the cells.^13^^,^^14^^,^^15^ We noted as previously reported that local anesthesia drugs could induce vacuolation.^5^ The vacuolation in human tooth pulp cells by the drugs is linked with autophagy that can be monitored by LC3II, a key autolysosomal marker.^6^ Using various approaches, we have shown that the autophagy marker LC3-II levels were increased in tooth pulp cells by local anesthetic drugs. Also by applying a LC3-GFP reporter assay, we showed that the LC3 protein was translocated into autophagosomes when primary tooth cells were treated with the anesthetic drugs.

Mechanistically, autophagy has often been linked with mitochondrial dysfunctions and localized inside mitochondria (i.e., also named as mitophagy).^16^ However, through detailed dynamic cellular energetic analysis we have identified that upon challenge by the different drugs tested, at least until 8 h after treatment, mitochondrial functions of tooth pulp cells were not diminished. Instead we have observed a significant increase of mitochondrial respiration, a possible surviving mechanism have been initiated in mitochondria to counteract the toxicity effect of the drugs. It is noticeable that longer treatment at high but not low concentration for most of the tested drugs did reduce basal respiration, phosphorylation and maximum respiration of mitochondria, in the meantime, unexpectedly the mitochondrial spare respiratory capacity remained unchanged (data not shown), suggesting at this stage and condition mitochondrial functions have been possibly damaged that require further experiments of checking mitochondria integrity and functions are desired.

Interestingly proton leak has been identified in all the tested drugs in all the conditions, suggesting it is a direct consequence of local anesthetic drug application. In fact, another local anesthetic drug bupivacaine has been found to have uncoupling function through a protonophore-like mechanism;^17^ therefore, it would be interesting to understand if articaine, mepivacaine and lidocaine function on proton leak using the same machinery. Continuous H vacuolar (V)-ATPase activity is required to maintain the pH in case of proton leak.^18^ Given the fact that acidic lysosomal condition is required for autophagosome maturation, we hypothesize that V-Atpase’s function has been overactivated upon drug challenge. Indeed, this hypothesis can be supported by the fact that adding V-Atpase specific inhibitor, bafilomycin could efficiently rescue cell proliferation. A future assay on V-Atpase activities would be helpful to further prove our theory. Also for later stage high-concentration drug treaments, bafilomycin failed to further induce LC3II accumulation also suggest that future tests need to be done on the other mechanisms involving in autophagy and dose effect of bafilomycin.

A key role of autophagy is to remove excess aggresomes associated p62 and dysfunctional organelles that can induce double strand DNA breaks.^18^ p62 is a receptor for cargo destined to be degraded by autophagy, including ubiquitinated protein aggregates.^19^ The p62 protein can bind both ubiquitin and LC3, thereby targeting ubiquitinated proteins to the autophagosome for clearance.^20^ In addition, p62 has been shown to regulate cell proliferation through Twist1 stabilization.^7^ Therefore, intracellular p62 protein levels are critical for cell viability. Mechanistically, using a signaling pathway specific PCR array, we identified the *p62* gene as a unique downstream target of all the drugs tested. We also found that the concentrations of anesthetic drugs are linked with p62 protein levels especially for later stages, while reduced cell proliferation by long time high-concentration local anesthetics treatment could not be rescued using bafilomycin. This observation suggests it is possible DNA damage could be induced by excess p62 when it reaches to a certain concentration and is not cleared in time.

In conclusion, our findings that local anesthetics can induce autophagy in tooth pulp cells have clinical implications, due to their potential impacts on tooth development as well as root formation and apical foramen closure. Future *in vivo* validation of our findings will be plausible to further enhance our knowledge about the clinical impacts of these local anesthetic drugs.

## Materials and methods

### Drugs

The commercial anesthetic drugs used in this study were articaine-based agents: Ubistesin (522721, 3 M ESPE), Ubistesin forte (512987, 3 M ESPE), Septanest (09091451103, Septodont) and mepivacaine-based agent: Scandonest (09091173002, Septodont) and Lidocaine based agent: Xylocaine (Batch 4180, Dentsply).

### Animal model of local anesthetics application

Freshly isolated mandibles from 5 months old Gloucester Old Spot crossed with Landrace pigs (kindly provided by a local abattoir) were used in the experiments. Local anesthetics: Lidocaine (for nerve block), Scandonest or Ubistesin–Forte (for infiltration) was prelabeled with 0.5% fluorescein. One milliliter Lidocaine was then injected into the mandibular foramen and 0.8 ml Scandonest or Ubistesin–Forte was injected respectively into buccal and lingual mesial submucous sites around the mesial apical root of mandibular first molars using 23G needle and 1-ml syringe. After 2 and 16 h, the third permanent molar tooth germ and the first permanent molar root pulp were excised and embedded in OCT compound (Tissue-Tek; Sakura Finetek, Thatcham, UK). Eight-micrometer frozen sections were prepared. The fluorescence images of the tissues were acquired using a Leica SP5 confocal microscope (Leica Microsystems (UK) Ltd, Breckland, UK) and measured with Adobe Photoshop CS6 software (Adobe Systems Europe Ltd, Maidenhead, UK) then normalized against the signal of the labeled drugs.

### Cell isolation and culture

Cell isolation and treatment protocols were approved by the Ethics Committee of the Peking University School and Hospital of Stomatology, Beijing, China. For details please see Supplementary Information.

### Autophagy Inhibitor assay

A 10-mM stock solution of the autophagy inhibitor bafilomycin A1 (No. 11707, Sigma) was prepared in DMSO. Cells were treated with 100 nM bafilomycin and control cells were treated with vehicle alone. For the autophagy inhibition assay, cells were exposed to anesthetics and bafilomycin at the same time.

### Plasmid preparation and transfection

GFP–LC3II plasmids^21^ were used for visualization of autophagosome formation. The GFP–LC3 fusion protein is expressed throughout the cytoplasm in the absence of autophagy, but translocates to the autophagosome membrane upon autophagy induction to form multiple bright green fluorescent spots. For details please see Supplementary Information.

### BrdU staining and quantification

BrdU (RPN201, Amersham-GE) was diluted in complete cell culture medium at a ratio of 1 : 1000 and added on top of tooth pulp cells for 2 h. Cells were then fixed in 4% PFA then treated with of 2 N HCl for 30 min before they were stained with anti-BrdU antibodies (ab6326, Abcam, 1 : 500 dilution). The antibody staining procedures were exactly as above except the blocking buffer was prepared with 2.5% BSA. After imaging, the images were processed in Image J software (National Institutes of Health, Bethesda, MD, USA) and BrdU-positive cells and total cell number were quantified using ‘Analyze Particles’ function.

### Terminal deoxynucleotidyl transferase dUTP nick end labeling (TUNEL) assay

Apoptosis was assayed using an In Situ Cell Death Detection Kit (11 684 795 910, Roche), following the manufacturer’s standard protocol.

### Immunostaining, western blotting real-time RT-PCR and result analysis

Immunostaining, western blotting, RNA and cDNA preparation, real-time RT-PCR, and statistical analysis were performed as previously described.^22^ For experimental details please see Supplementary Information.

### PCR array

Changes in human signal transduction genes were measured using an RT^2^ Profiler PCR Array (Human Signal Transduction PathwayFinder; PAHS-014ZG-4, Qiagen Ltd., Manchester, UK) on a Lightcycler 480 Instrument II (Roche Diagnostics Limited, Burgess Hill, UK) 384-well block real-time PCR machine according to the manufacturer’s instructions. 1 μg cDNA template was used for each sample. Results were exported into Microsoft Excel and heatmap analysis was performed using Multiple Experiment Viewer (MeV) 4.9.0 open source software (Dana-Farber Cancer Institute, Boston, MA, USA).

### Mitochondrial energetic assay

The XF Cell Mito Stress Test (#103015-100, Seahorse Bioscience, Copenhagen, Denmark) was used in XF 96 Extracellular Flux Analyzer (Seahorse Bioscience) to measure key parameters of mitochondrial function by directly measuring the oxygen consumption rate (OCR) of live cells. For details please see Supplementary Information.

### Statistical analysis

PRISM 5 software (GraphPad Software, Inc. La Jolla, CA, USA) was used to analyze the experimental data. One-way ANOVA followed by Bonferroni correction was performed for real-time RT-PCR analysis, and Dunnett’s test was applied for mitochondrial energetic analysis. Statistical significance was set at *P<0.05 and **P<0.01.

## Abbreviations

36β4: ribosomal protein, large, P0
ACSL5: acetyl-CoA synthetase long-chain family member 5
ADM: adrenomedullin
ARNT: aryl hydrocarbon receptor nuclear translocator
ATG3: autophagy related 3
ATG5: autophagy related 5
ATG7: autophagy related 7
ATG12: autophagy related 12
Bax: B-cell chronic lymphocytic leukemia/lymphoma 2-associated X protein
BBC3: B-cell chronic lymphocytic leukemia/lymphoma 2 binding component 3
BCL2: B-cell chronic lymphocytic leukemia/lymphoma 2
BCL2A1: B-cell chronic lymphocytic leukemia/lymphoma 2-related protein A1
BIRC3: baculoviral inhibitor of apoptosis protein repeat containing 3
BMP2: bone morphogenetic protein 2
BMP4: bone morphogenetic protein 4
BrdU: bromodeoxyuridine
BSA: bovine serum albumin
CCL5: chemokine (C–C motif) ligand 5
CCND2: cyclin D2
CDKN1A: cyclin-dependent kinase inhibitor 1A
CDKN1B: cyclin-dependent kinase inhibitor 1B
cDNA: complementary DNA
CEBPD: CCAAT/enhancer binding protein, delta
CPT2: carnite palmitoyltransferase 2
CSF1: colony-stimulating factor
DAB2: DAB, mitogen-responsive phosphoprotein homolog 2
DAPI: 4′,6-diamidino-2-phenylindole dilactate
DMSO: dimethyl sulfoxide
EGFR: epidermal growth factor receptor
EMP1: epithelial marker protein 1
FCER2: Fc fragment of imuloglobulin E, low-affinity II receptor
FPSL1: farnesyl diphosphate synthase pseudogene 1
GATA3: GATA-binding protein 3
GCLC: glutamate-cysteine ligase, catalytic subunit
GCLM: glutamate–cysteine ligase, modifier subunit
GFP: green fluorescent protein
GFP–LC3: green fluorescent protein fused microtubule-associated protein 1 light chain 3 alpha
GSR: glutathione reductase
HCl: hydrochloric acid
HMOX1: heme oxygenase I
ICAM1: intercellular adhesion molecule 1
ID1: Inhibitor of DNA Binding 1
IFNG: Interferon Gama
Ki67: marker of proliferation Ki67
LAMP-1: lysosomal-associated membrane protein 1
LC3: microtubule-associated protein 1A/1B-light chain 3
LC3 I: microtubule associated protein 1 light chain cytosolic form
LC3 II: microtubule-associated protein 1 light-chain membrane-bound form
Lido: lidocaine
MCL1: myeloid cell leukemia 1
MeV: multiple experiment viewer
MMP7: matrix metallopeptidase 7
mTor: mechanistic (or mammalian) target of rapamycin
MYC: myelogtomatosis oncogene
NQO1: nicotinamide adenine dinucleotide phosphate dehydrogenase quinone1
OCR: oxygen consumption rate
OCT: optimum cutting temperature
OLR1: oxidized low density lipoprotein (lectin-like) receptor 1
P38: mitogen-activated protein kinase 14
P62: sequestosome 1
PFA: paraformaldehyde
p-mTOR: phosphor-mechanistic (or mammalian) target of rapamycin
PPARD: peroxisome proliferator-activated receptor delta
PTCH1: Patched 1
RT-PCR: real-time PCR
Runx2: runt-related transcription factor 2
Scan: Scandonest
Sep: Septanest
SLC27A4: solute carrier family 27 (fatty acid transporter), member 4
SLC2A1: solute carrier family 2 (facilitated glucose transporter), member 1
SOCS3: suppressor of cytokine signaling 3
SQSTM1 (P62): Sequestosome 1
STAT1: signal transducer and activator of transcription 1
TNF: tumor necrosis factor
TNFSF10: tumor necrosis factor (ligand) superfamily, member 10
TUNEL: terminal deoxynucleotidyl transferase dUTP nick end labeling
Twist1: twist family BHLH transcription factor 1
TXN: Thioredoxin
TXNRD1: thioredoxin reductase 1
UBI: Ubistesin
UBI-F: ubistesin forte
V-ATPase: vacuolar-type H^+^–ATPase
VEGFA: vascular endothelial growth factor A
WNT2B: wingless-type MMTV integration site family, member 2B
WNT3A: wingless-type MMTV integration site family, member 3A.
