# Supplementary material for: Local anesthetics induce autophagy in young permanent tooth pulp cells
Source: Cell Death Discov. 2015 Sep 7;1:15024–. doi: 10.1038/cddiscovery.2015.24 (PMC4979463; doi:10.1038/cddiscovery.2015.24)
Supplement: Supplementary Materials [file cddiscovery201524-s1.pdf]

Cell isolation and culture

Specifically, young permanent tooth pulp cells were obtained from two patients (12 and 16 years old) whose upper first premolars were extracted for orthodontic reasons. Cell isolation and treatment protocols were approved by the Ethics Committee of the Peking University School and Hospital of Stomatology, Beijing, China. Specifically, young permanent tooth pulp cells were obtained from two patients (12 and 16 years old) whose upper first premolars were extracted for orthodontic reasons. Following extraction, the tooth chamber was first opened using a dental drill. Pulp tissue was isolated using a 23-gauge fine needle and digested with 3% collagenase I (C0130-1G; Sigma) dissolved in Hank's balanced salt solution (Gibco, Cat. No.14175-053) supplemented with 1% penicillin-streptomycin (SV30079.01, Hyclone) using a final volume of 2ml enzyme solution per gram of tissue for 1 hour at 37°C with constant agitation. The enzyme reaction was then stopped by adding an equal volume of complete cell culture medium, comprising Dulbecco modified Eagle's medium/F12 (31331-028, Gibco) supplemented with 20% fetal bovine serum (F7524, Sigma) and 1% penicillin-streptomycin. The culture medium was changed every 2 days and cells were passaged when they reached 70–80% confluence by digestion with 0.05% Trypsin-EDTA (25300-054, Gibco). Passage 7–9 cells at 70% confluence were used for all experiments.

Plasmid preparation and transfection

Plasmids were amplified and extracted using Plasmid Maxi Kit (12162, Qiagen), according to the manufacturer's recommendations. Cells were seeded at $2x10^5$ cells/ml into 6-well plates and cultured for 24 hours until 60–80% confluence. For

each transfection, 2μg of plasmid DNA was mixed with 200μL of jetPRIME buffer (114-15, PolyPlus) and the mixture was added to an equal volume of jetPRIME solution and added to cells. Transfected cells were treated with anesthetic drug treatment after 24 hours.

Immunostaining

Cells cultured on glass coverslips were fixed in 4% ice-cold *paraformaldehyde (diluted in 10mM PBS),* incubated at room temperature for 20–30 minutes, and then rinsed three times for 5 min in PBST (10mM PBS containing 0.1% Triton-X100). Non-specific binding sites were first blocked by incubation in PBST containing 5% donkey serum, 0.25% cold water fish gelatin and 0.25% BSA for 60 minutes. Immunofluorescence images were obtained using either a Leica SP5 or a Zeiss LSM510Meta laser-scanning microscope. For antibody details please see Supplemental Information.

Primary antibodies were anti-LC3A/B (D3U4C; Cell Signaling, Cat. No. 12741; 1:1000 dilution), and anti-lysosome-associated membrane glycoprotein 1 ([LAMP-1] D2D11; 9091, Cell Signaling; 1:250 dilution). These were diluted in blocking buffer and incubated overnight at 4°C with cells. After three 5 minute washes in PBST, cells were incubated with fluorochrome-conjugated secondary antibodies diluted in blocking buffer for 1–2 hours at room temperature in the dark. Secondary antibodies were Alexa 488 donkey anti-rabbit IgG (A21206, Life Technologies) and Alexa 568 donkey anti-rabbit IgG (A10042, Life Technologies). Cells were counterstained with 4′,6-diamidino-2-phenylindole (DAPI, D9542, Sigma-Aldrich, 1:10000) for 10

minutes. The cytoskeleton was visualized by counterstaining with DyLight 554 Phalloidin (13054, Cell Signaling, 1:200 dilution).

Western blotting

Proteins were extracted using Ripa buffer (89901, Pierce) supplemented with Halt Protease and a Phosphatase Inhibitor Cocktail (78440, Pierce), and quantified using a BCA Protein Assay (23225, Pierce). Protein separation and membrane transfer were performed using NuPage precast gels (NP0335BOX, Life Technologies) and transfer buffer (NP0321, Life Technologies). Antibody incubation and washes were performed using the iBind system (Life Technologies). Anti-LC3A/B (D3U4C; 12741, Cell Signaling, 1:5,000), anti-p62 (PM045, MBL International, 1:3,000) and anti-phospho mTor (Ser2481) (2974, Cell Signaling, 1:2,000) primary antibodies were used. As a loading control, blots were simultaneously probed with anti-b-actin antibodies (8457P, Cell Signaling, 1:5,000) or re-probed after treating with Restore Western Blot Stripping Buffer (21059, Pierce). Protein bands were visualized using a C-DiGit system (Licor) with C-Digit Image Studio (Version: 1.0.19, Licor) software. Detected molecular weight: LC3I: ~19 kDa; LC3II: ~17 kDa; b-actin: 45 kDa; p62: 62kDa; pmTor: 289kDa.

Real-time RT-PCR and result analysis

RNA and cDNA preparation, real-time RT-PCR, and statistical analysis were performed as previously described (Hu et al., 2012). Primers to the human genes used in this study were *Atg3-F*, 5′-TTTGGCTATGATGAGCAACG-3′; *Atg3-R*, 5′-AAGTTCTCCCCCTCCTTCTG-3′; *Atg5-F*, 5′-CAGATGGACAGTTGCACACA-3′; *Atg5-R*, 5′-CTGTTGGCTGTGGGATGATA-3′; *Atg7-F*, 5′-CTGGGGACTTGTGTCCAAAC-3′; *Atg7-R*, 5′-AGAGGTTGGAGGCTCATTCA-

3′; *Atg12-F*, 5′-AATCAGTCCTTTGCTCCTTCC-3′; *Atg12-R*, 5′-CACGCCTGAGACTTGCAGTA-3′; *Bax-F*, 5′-GGCATCATTAACTGGGGAAG-3′; *Bax-R*, 5′-TCCAGCCAGATTTAGGTTCAA-3′; Beclin-F, 5′-AGGTTGAGAAAGGCGAGACA-3′; Beclin-R, 5′-AATTGTGAGGACACCCAAGC-3′; *Ki67*-F, 5′-GAATTGAACCTGCGGAAGAG-3′; *Ki67*-R, 5′-TTTGCTGTTCTGCCTCAGTC-3′; *LC3-F*, 5′-CGTCCTGGACAAGACCAAGT-3′; *LC3-R*, 5′-TCCTCGTCTTTCTCCTGCTC-3′; *p38-F*, 5′-GTCAACTGGAGCAAGAAGGA-3′; *p38-R*, 5′-ATGTGGTCACATGTGCAAAG-3′; *p62-F*, 5′-TGGACCCATCTGTCTTCAAA -3′; *p62-R*, 5′-ATGGACAGCATCTGGGAGAG-3′; *Runx2-F*, 5′-AAATGCTGGAGTGATGTGGT-3′; and *Runx2-R*, 5′-TATGAAGCCTGGCGATTTAG-3′.

Mitochondrial energetic assay

The XF Cell Mito Stress Test (#103015-100, Seahorse Bioscience) was used in XF 96 Extracellular Flux Analyzer (Seahorse Bioscience) to measure key parameters of mitochondrial function by directly measuring the oxygen consumption rate (OCR) of live cells. The materials used included: XFe96 FluxPak (Seahorse Bioscience), XF96 Cell Culture Microplates (Seahorse Bioscience), XF Calibrant (Seahorse Bioscience), XF Base Medium (Seahorse Bioscience), Sodium Pyruvate (100mM, Sigma S8636), L-Glutamine (G-5763, Sigma), Glucose (1.0 M, Sigma G8769).

**Compound(s)**	**ETC target**	**Effect on OCR**
Oligomycin	ATP Synthase (Complex V)	Decrease
FCCP	Inner Mitochondrial Membrane	Increase
Rotenone/ antimycin A	Complex I and III	Decrease

(respectively)

Compounds used in XF Cell Mito Stress Test

Firstly, optimal concentrations of three compounds (final concentration 1uM) and cell seeding density were empirically determined prior to the assay. Cells were seeded at 10×10^3 cells/well in 100 µl of DMEM/F12 with 20%FBS in XF96 Cell Culture Microplates using 8 replicates and incubated for 24 hours at 37 °C in 5% CO^2 atmosphere. Five local anesthetics of 0.5mM and 2mM concentration were added with one control group. After 2/4/8/16 hours, the microplates were ready for check in the machine. The drug injections ports of the XF Assay Cartridge were loaded with the assay reagents in assay medium. 25 µl of the three compounds were added sequentially. Culture medium was exchanged with assay medium prior to measurements. Culture medium was aspirated and 80 µl pre-warmed assay medium added twice, aspirated and 175 µl pre-warmed assay medium added. The microplate was equilibrated in a CO^2 free incubator at 37ºC for 60 minutes. During this equilibration period, the XF96 Analyzer was calibrated with a calibration plate that had been hydrated at 37ºC overnight using the standard XF calibration protocol. Following calibration, the calibration plate was replaced with the XF96 cell culture microplate containing pre-treated cells with local anesthetics and the experimental run started. Data were normalized by cell number and expressed as pmol of O^2 per minute per 10^ 4 cells.
