# Supplementary figures and images for: Local anesthetics induce autophagy in young permanent tooth pulp cells
Source: Cell Death Discov. 2015 Sep 7;1:15024–. doi: 10.1038/cddiscovery.2015.24 (PMC4979463; doi:10.1038/cddiscovery.2015.24)

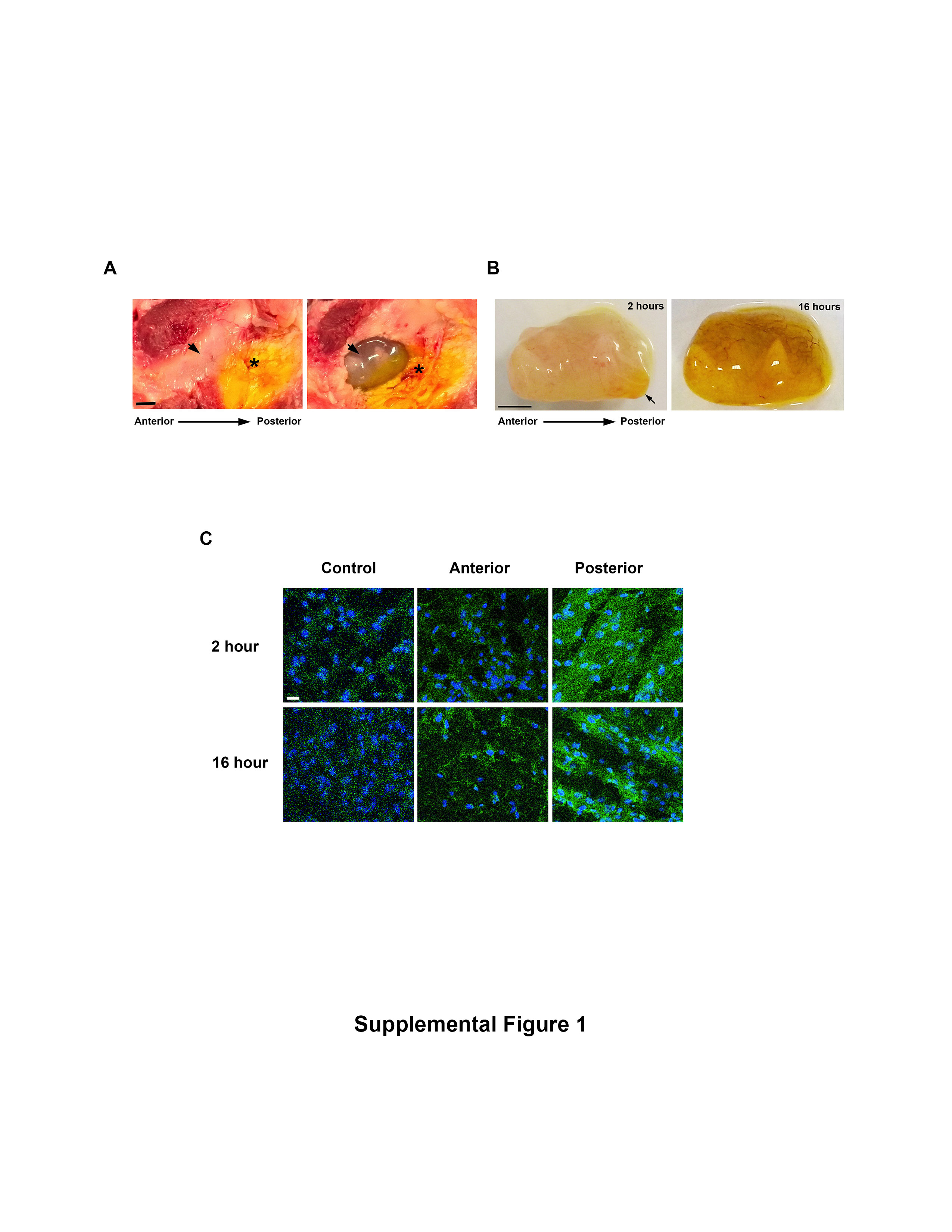

Supplement: Supplementary Figure S1-1 [file cddiscovery201524-s2.jpg]

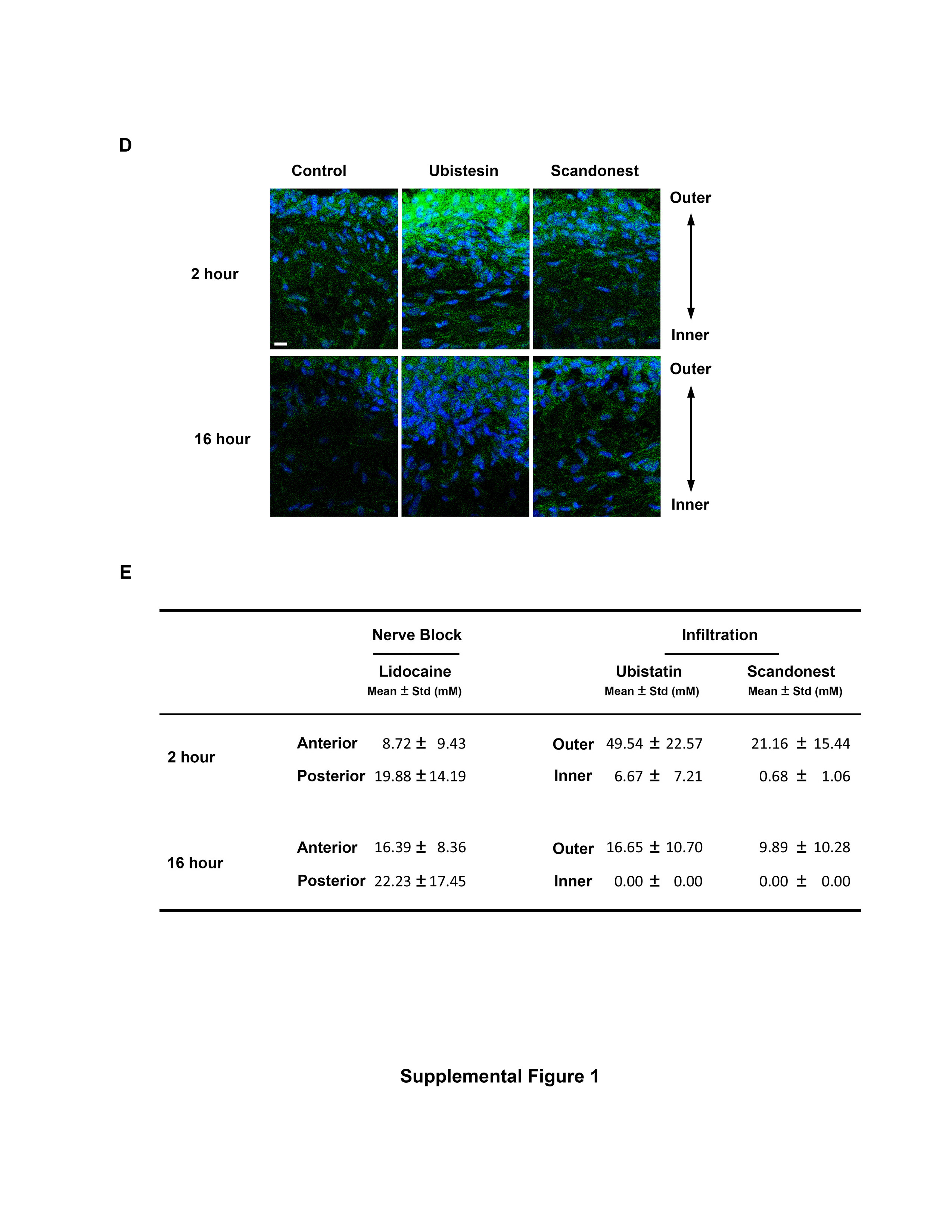

Supplement: Supplementary Figure S1-2 [file cddiscovery201524-s3.jpg]

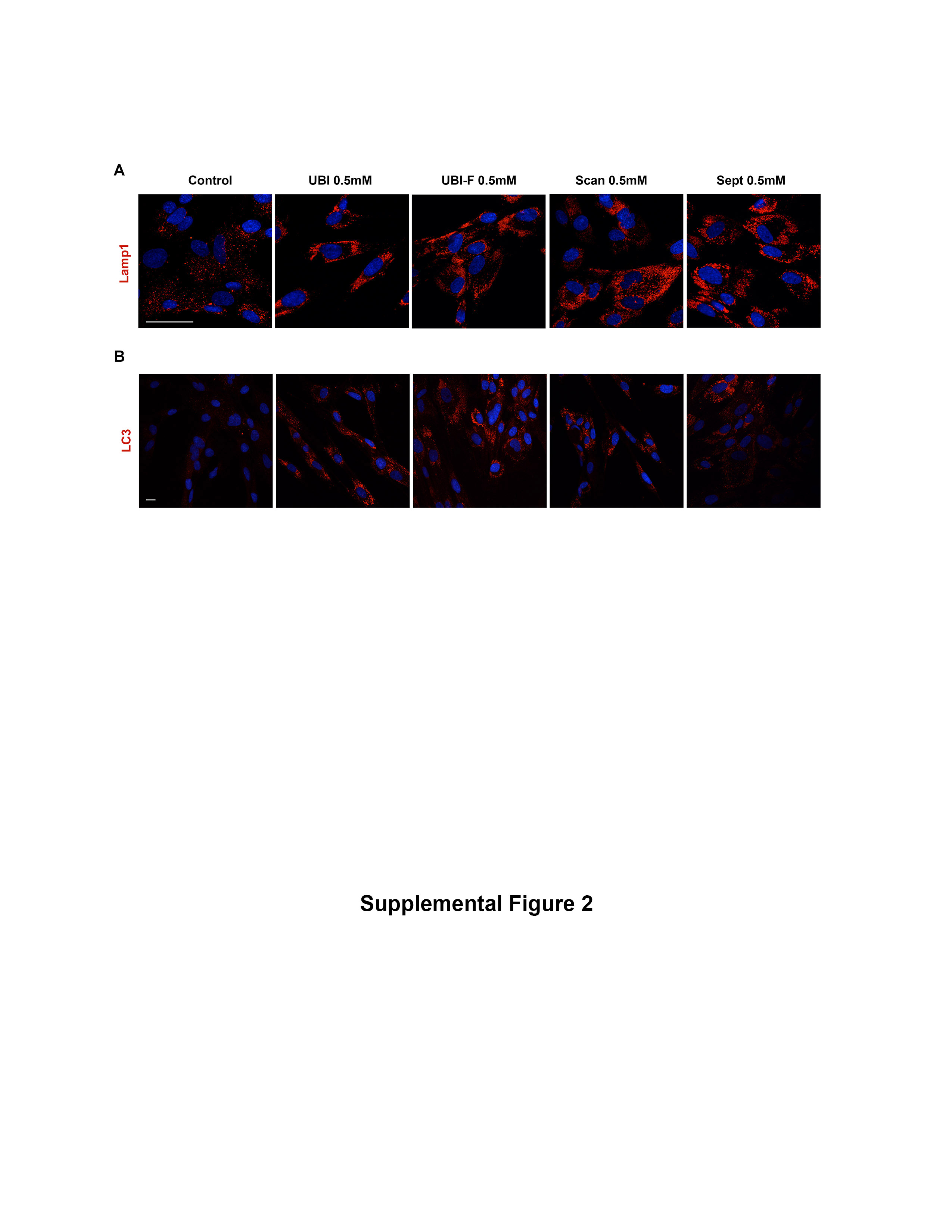

Supplement: Supplementary Figure S2-1 [file cddiscovery201524-s4.jpg]

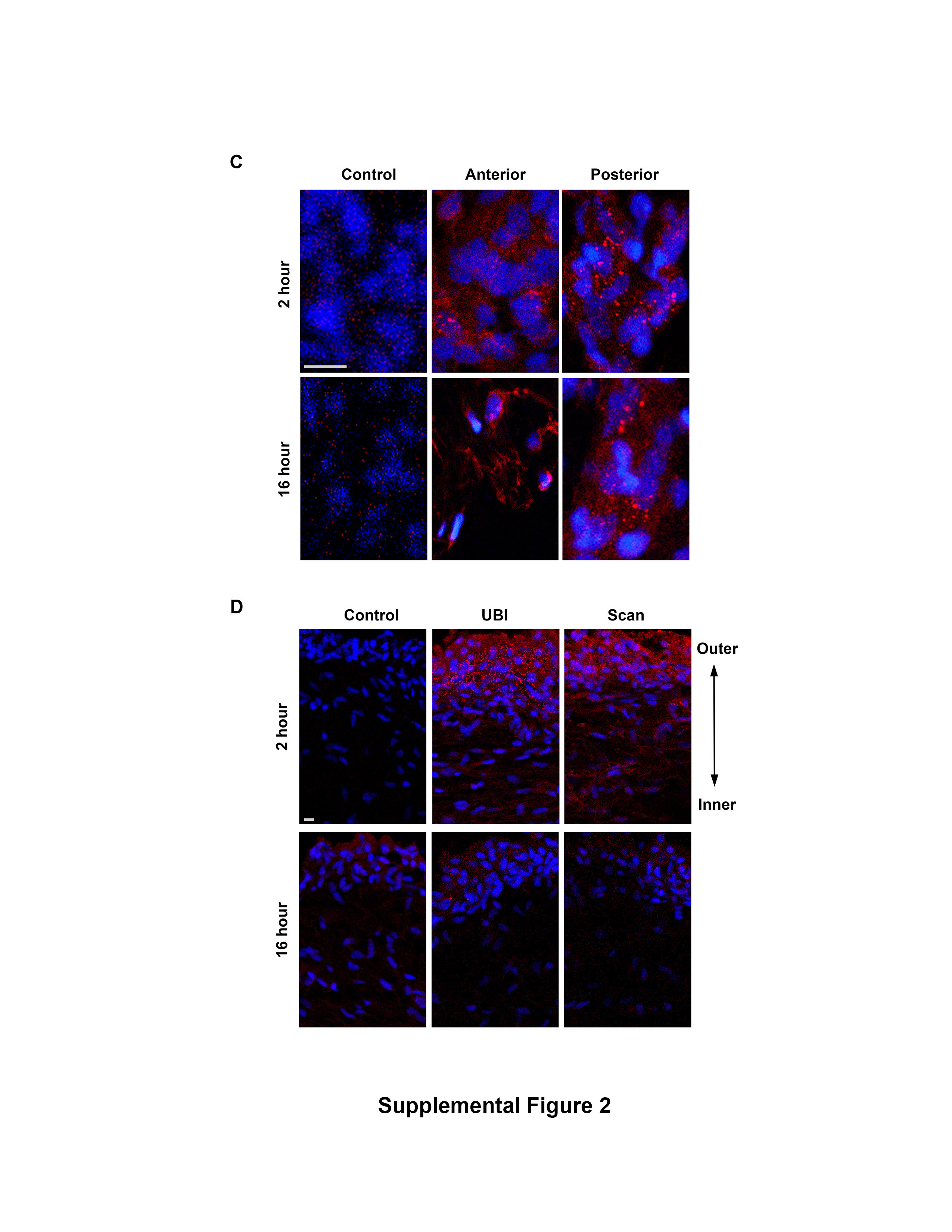

Supplement: Supplementary Figure S2-2 [file cddiscovery201524-s5.jpg]

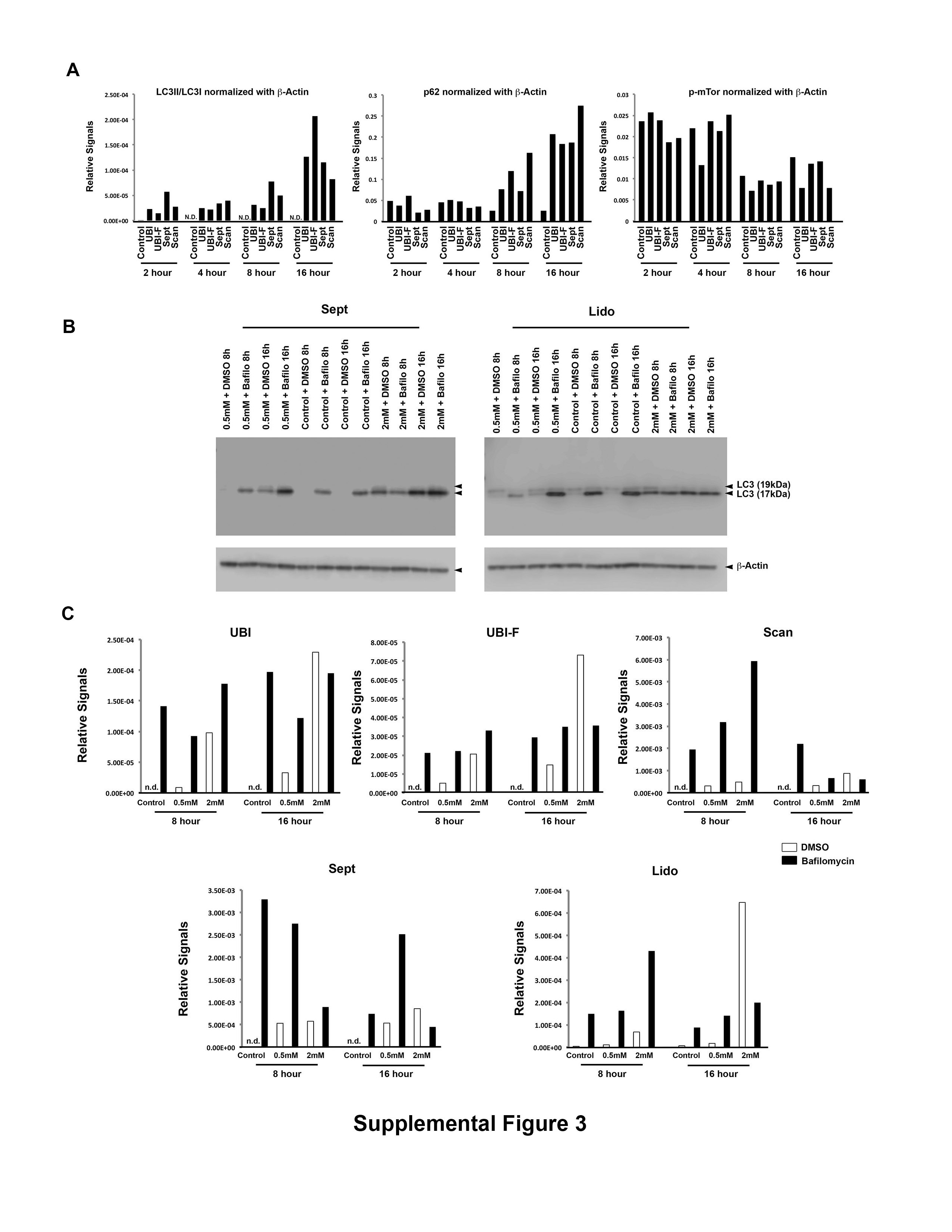

Supplement: Supplementary Figure S3 [file cddiscovery201524-s6.jpg]

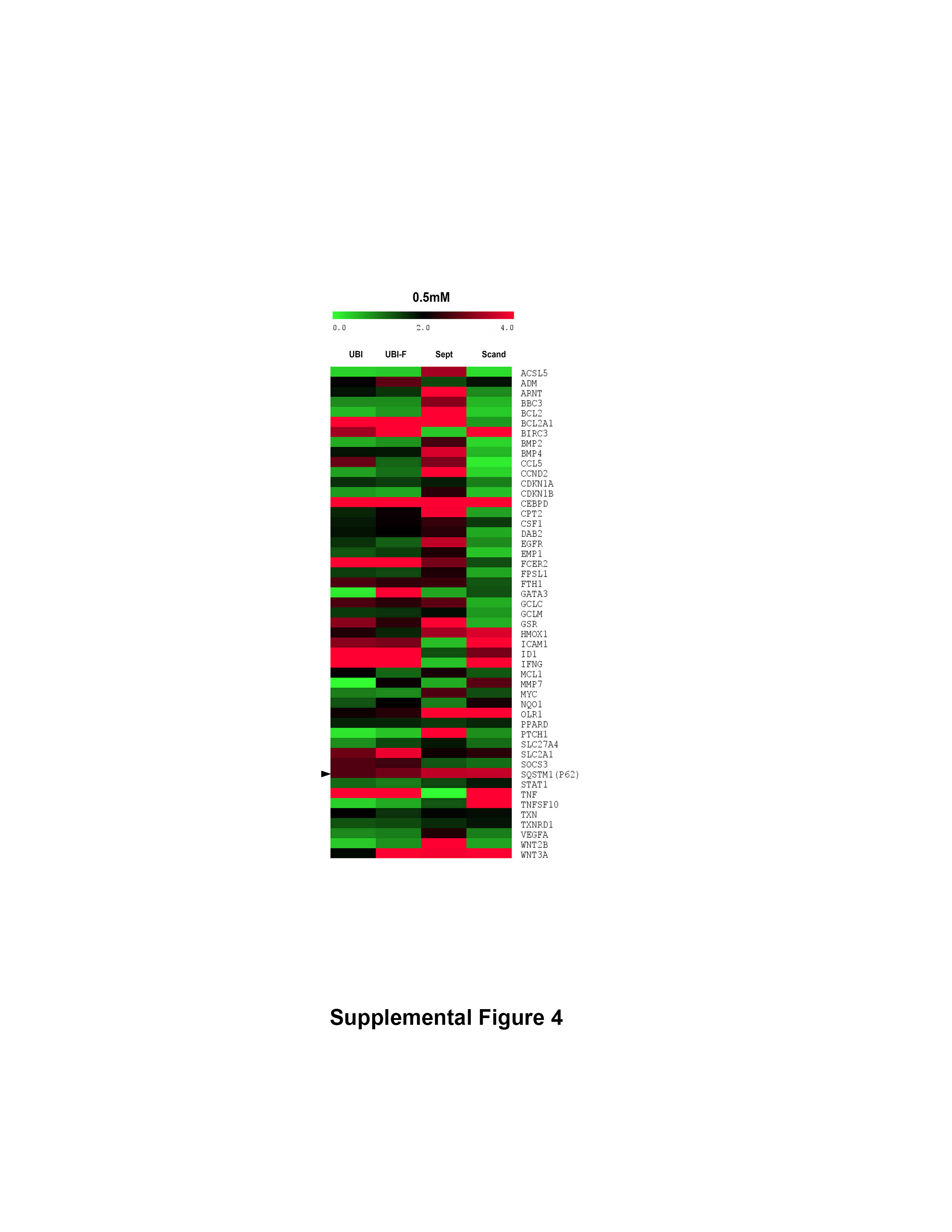

Supplement: Supplementary Figure S4 [file cddiscovery201524-s7.jpg]

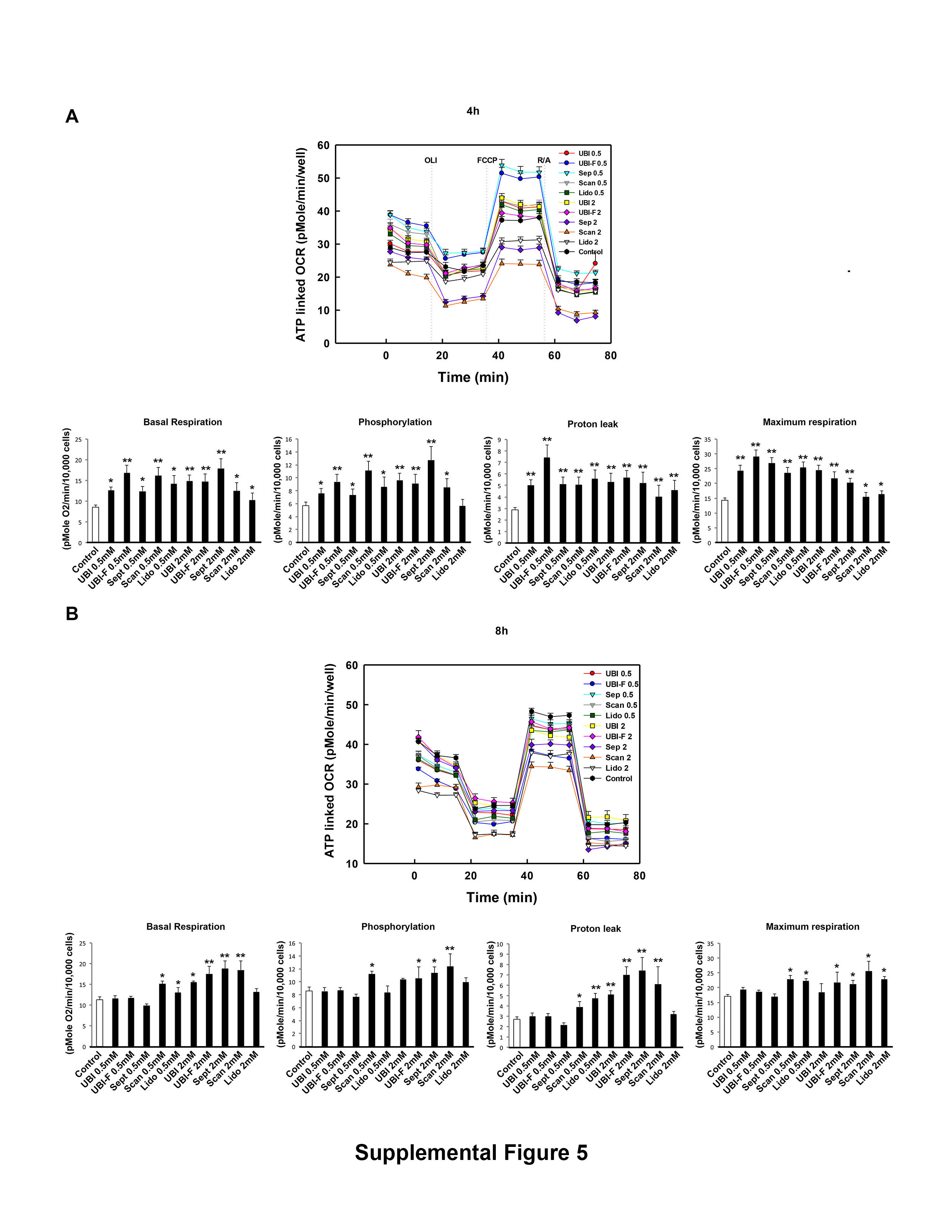

Supplement: Supplementary Figure S5 [file cddiscovery201524-s8.jpg]
