# Supplementary material for: Local anesthetics induce autophagy in young permanent tooth pulp cells
Source: Cell Death Discov. 2015 Sep 7;1:15024–. doi: 10.1038/cddiscovery.2015.24 (PMC4979463; doi:10.1038/cddiscovery.2015.24)
Supplement: Supplementary Figure Legends [file cddiscovery201524-s9.doc]

**SUPPLEMENTARY LEGENDS FOR FIGURES**

**Supplemental Figure 1. Local anesthetics can infiltrate into tooth germ and pulp.**

**A.** Fluorescein labelled lidocaine was injected into freshly extracted 5-months-old pig mandible through foramen (asterisk) for 2 hours. The bone chamber containing the third lower permanent molar tooth germ was indicated with arrow (left panel). The tooth germ was then exposed to illustrate the close anatomical association of the tooth with mandibular foramen and canal (right panel). Note that the drug (represented by yellow color visually) has already entered the tooth socket and stained the tooth. **B**. After 2 and 16 hours, the third molar germs were extracted and examined. The color difference indicates where and how much of the anaesthetics infiltrate into the different compartments of the tooth germs. **C.** Representative images for the fluorescein labelled lidocaine (green color) distribution inside the 3rd molar germs after receiving lidocaine nerve block injection. **D**. Representative images of the apical part of the first molar mesial root pulp at 2 and 16 hours after infiltration injection with fluorescein labelled Ubistetin and Scandonest (green color). **E**. The concentration of the drugs were analysed by measuring green fluorescence intensity using a Leica SP5 confocal microscope and Photoshop CS6 software, and calculated in Prism 5.0 software at random different sites of the tooth pulp of the third molar pulp (for nerve block) and the first molar mesial root pulp (for infiltration). The values represent average signals from 4 independent samples. Cell nuclei were visualized using 4′,6-diamidino-2-phenylindole (DAPI). Scale bars, 10μm.Scale bars, 5mm.

**Supplemental Figure 2. Local anesthetics induce autophagy in tooth pulp cells.**

Immunofluorescence analysis of Lamp-1 (red) (**A**) and LC3 (red) (**B**) expression following treatment with 0.5mM anesthetic drug. **C** and **D,** Immunofluorescence analysis of LC3 (red) expression in 2-hour and 16-hour samples as indicated in **Supplemental Figure 1** for nerve block (**C**) and infiltration injections (**D**). Cell nuclei were visualized with DAPI. UBI, Ubistesin; UBI-F, Ubistesin forte; Scan, Scandonest; Sept, Septanest. Scale bars, 10μm.

**Supplemental Figure 3. Local anesthetics affect tooth pulp cell proliferation via autophagy induction.**

**A**. Quantification of the western blot experiments in **Figure 3B**. For LC3, LC3II/LC3I ratio and normalized with β-Actin signal. For p62 and phosphor-mTor the signal were normalized directly to β-Actin. **B**. Western blot analysis of LC3 protein levels in cells treated with 0.5mM and 2mM concentrations of the indicated anesthetic drugs and together with 100nM bafilomycin for 8 and 16 hours. β-actin was used as a loading control by stripping and re-blotting the same blot. The blots are from the same series of experiment showed in **Figure 3C**. **C**. Quantification of the western blot experiments in **Figure 3C** and **Supplemental Figure 3B**. For LC3, LC3II/LC3I ratio and normalized with β-Actin signal. Lido, Lidocaine; UBI, Ubistesin; UBI-F, Ubistesin forte; Scan, Scandonest; Sept, Septanest.

**Supplemental Figure 4. *p62* is the common downstream target of anesthetic drugs on tooth pulp cells.**

Heatmap analysis of PCR array data showing changes in gene expression in tooth pulp cells after treatment with 0.5 mM anesthetic drug (for 2mM dosage data, please see **Figure 4A**). Colors of the bars reflect fold changes in expression, as indicated by the key.

**Supplemental Figure 5. Local anesthetics affect tooth pulp cellular energetics.**

Key parameters of mitochondrial function including basal respiration, phosphorylation, proton leak and maximum respiration were tested by directly measuring the oxygen consumption rate (OCR) of cells treated with the five anesthetics of 0.5mM and 2mM concentrations for 4 (**A**) and 8 (**B**) hours. 2 hours and 16 hours results can be found in **Figure 5A and B**. Lido, Lidocaine; UBI, Ubistesin; UBI-F, Ubistesin forte; Scan, Scandonest; Sept, Septanest. Number abbreviations after individual drug abbreviation: 0.5: 0.5mM; 2: 2mM. * p<0.05; ** p<0.01.
